# What Do Primary Healthcare Providers and Complementary and Alternative Medicine Practitioners in Palestine Need to Know about Exercise for Cancer Patients and Survivors: A Consensual Study Using the Delphi Technique

**DOI:** 10.1155/2019/7695818

**Published:** 2019-04-17

**Authors:** Ramzi Shawahna, Mahmoud Al-Atrash

**Affiliations:** ^1^Department of Physiology, Pharmacology and Toxicology, Faculty of Medicine and Health Sciences, An-Najah National University, Nablus, State of Palestine; ^2^An-Najah BioSciences Unit, Centre for Poisons Control, Chemical and Biological Analyses, An-Najah National University, Nablus, State of Palestine; ^3^Department of Physical Education, Faculty of Educational Sciences and Teachers' Training, An-Najah National University, Nablus, State of Palestine

## Abstract

**Background:**

Exercise has physiologic and psychological benefits for cancer patients and survivors. Today, various exercises are recommended as adjunct to therapies for cancer patients and survivors. This study was conducted to develop a consensual core list of important knowledge items that primary healthcare providers and complementary and alternative medicine (CAM) practitioners need to know on the role of exercises and physical activities in stimulating anticancer immunity.

**Methods:**

Knowledge items were collected following interviews with key contact experts (4 oncologists, 3 exercise and medicine specialists, 2 researchers, 2 cancer patients, and 3 survivors) and extensive literature review. The collected knowledge items were rated by 9 researchers who conducted research on exercise and cancer. A modified two-iterative Delphi technique was employed among a panel (*n = 65*) of healthcare providers and CAM practitioners to develop the consensual core list of knowledge items.

**Results:**

Of the 49 knowledge items, consensus was achieved on 45 (91.8%) items in 6 categories. Of those, 9 (20.0%) were general items on recommending moderate to vigorous habitual exercises and physical activities. The rest of items were related to the effects of habitual exercises and physical activities on the functions of immune system and exposure to carcinogens 16 (35.6%), anticancer therapies 12 (26.7%), metastasis of cancer 3 (6.7%), metabolism within tumors 3 (6.7%), and myokines release 2 (4.4%).

**Conclusion:**

Formal consensus was achieved for the first time on a core list of knowledge items on how exercises and physical activities might stimulate anticancer immunity. This core list might be considered at the time of developing training/educational interventions and/or continuing education for primary healthcare providers and CAM practitioners. Future studies are still needed to investigate if such consensual lists might improve congruence in cancer care continuum and improve survival rates and wellbeing of cancer patients and survivors.

## 1. Introduction

Cancer is a global major public health concern that will be placed at the top of the list of causes of death in the 21^st^ century [1]. The American Cancer Society estimates that there will be 1,735,350 new cancer cases and 609,640 cancer–related deaths in the US during the year 2018 [2]. Globally, there will be 18.1 million new cancer cases and 9.6 million cancer–related deaths in the year 2018 according to the estimates of the International Agency for Research on Cancer [3]. Incidence of cancer is on the rise globally. The risk of developing cancer in one's life was estimated at 50% in the UK [4]. However, it is noteworthy mentioning that survivorship is on the rise too. The Dutch Cancer Society estimates a 61% increase in survival rate of patients with cancer between 2010 and 2020 [5, 6].

Traditionally, oncologists in tertiary care hospitals have provided the majority of healthcare to and followed up with cancer patients and survivors [7]. With the increasing number of patients and survivors, a shortage of a sustainable oncologist-based model of care was predicted [8]. Alternatively, primary care-based management and followups were suggested to assume a greater role in catering to the needs of cancer patients and survivors. Today, diagnosis of cancer in primary healthcare practice is relatively common. In the UK, a primary healthcare provider typically sees about 70 cancer patients and this number is expected to increase by 2-fold by the year 2040 [9, 10]. In previous studies, primary healthcare providers reported heavy involvements in providing general medical care to patients with cancer, provided therapies, and helped patients make decisions [10–13]. Similarly, studies from different regions of the world have shown that a considerable number of cancer patients and survivors consult complementary and alternative medicine (CAM) practitioners [14–16]. Both primary healthcare providers and CAM practitioners are well placed within the community to provide holistic healthcare delivery and are more likely to maintain contact with cancer patients and survivors. Similarly, primary healthcare providers and CAM practitioners are accessible and trusted source of information for cancer patients and survivors [17, 18]. Grunfeld et al. reported that cancer patients were more satisfied when they were followed up in primary care compared to specialist care [19]. Enhanced patient support, more proactive care, and better teamwork between different disciplines of healthcare providers were cited as additional benefits of consulting primary healthcare providers and CAM practitioners [10, 14–16, 20].

Conventional treatments using chemotherapeutic agents are associated with significant side effects that limit satisfaction with and adherence to these therapeutic modalities [14]. Additionally, many patients are diagnosed at an advanced stage of cancer; subsequently, conventional therapies might be less effective in managing their cancers. As a result, cancer patients and survivors are increasingly seeking alternative or additional modalities such as CAM [21]. Today, CAM modalities are either used as complementary or alternatives to conventional therapies [14].

Many forms of exercise have evolved as promising CAM modalities that many cancer patients and survivors use as adjuvant therapies to complement conventional therapies, to stimulate their immunity against cancer, and/or to suppress cancerous cells. Some of these modalities have shown promising outcomes in animal,* in vivo*, and/or* in vitro* models [22–26]. A growing body of research has shown that certain exercise was beneficial in stimulating anticancer immunity and improved survival rates of cancer patients [22, 23, 27–29]. The Clinical Oncology Society of Australia has recommended that all patients with cancer should be prescribed exercise as an integral part of their therapy regimen [30]. This position statement which was endorsed by 25 leading healthcare organizations echoed recommendations by other health organizations in the US and UK like the American College of Sports Medicine and Cancer Research UK which recommended exercise as adjunct to cancer therapy to reduce adverse effects of the disease and its therapy and improve the quality of life of the patients. With advancement of modern healthcare provision, patients are increasingly informed with all treatment options and practices that have the potential to improve their survivorship and quality of life [31]. It has been argued that well informed patients experience better quality of life, cope with adverse effects of the therapy, and avoid overestimating the benefits of the therapeutic option [32].

Despite the growing body of evidence on the potential benefits of exercise on stimulating anticancer immunity and suppression of cancerous cells, little was narrated on what knowledge items explaining how exercise might stimulate anticancer immunity and suppress cancerous cells that primary healthcare providers and CAM practitioners need to be equipped with to expand their roles in cancer control and supporting shared decisions with their patients. Cancer education has been recognized as underdeveloped in medical schools, specialty training, continuous education, and professional development [33–35]. To bridge gaps in knowledge, recommended cancer curricula have been developed for medical students in different countries [35–37].

Currently, a core list of knowledge items that primary healthcare providers and CAM practitioners in Palestine or elsewhere need to know on how exercise and physical activities might stimulate anticancer immunity is lacking. Therefore, this study was conducted to achieve formal consensus among primary healthcare providers and CAM practitioners on a core list of knowledge items that primary healthcare providers and CAM practitioners in Palestine need to know on how exercise might stimulate anticancer immunity and suppress cancerous cells.

## 2. Methods

### 2.1. Design of the Study

**Figure 1:**
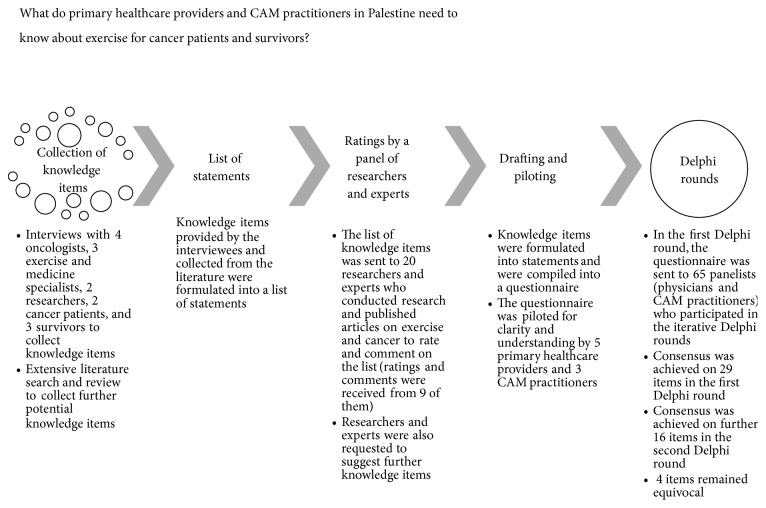
A flow diagram illustrating the different stages of the study.

In this study, we used a modified Delphi technique to achieve consensus among a panel of experts on a core list of knowledge items related to the role of exercise in stimulating anticancer immunity and suppression of cancerous cells that primary healthcare providers and CAM practitioners should know to enrich their knowledge and expand their understanding of how exercise related physiologic and endocrine changes might impact occurrence of cancer and enhance therapeutic agents used to treat cancer.  Figure 1 shows a flow diagram illustrating the different stages of the study.

Since its inception, the Delphi technique has evolved as a tool combining qualitative and quantitative approaches to achieve consensus among a panel of participants with prior knowledge of a topic being investigated [32, 38–41]. The Delphi technique was commonly employed for developing concepts and reaching consensus on issues when limited or no consensus exists. The technique was used extensively in achieving consensus on issues related to healthcare. Compared to other formal consensus methods like nominal, focused groups and round-table meetings, the Delphi technique has a number of appealing merits. The merits of the Delphi technique include overcoming geographical barriers when attempting to recruit participants to the panel of experts, save the costs of travel and bringing the panelists to a meeting, reserving the anonymity of the panelists, counteracting the possibility of one participant or a group of participants dominating the discussion and voting processes [32, 38–42]. In the Delphi technique, it is imperative that the panelists possess prior knowledge of the subject being investigated. The panelists are provided with questionnaires containing a series of statements. The panelists are requested to vote either by agreement or disagreement on each statement. Iterative rounds are repeated over and over again during an extended period of time until consensus is achieved [38, 42]. The panelists are commonly encouraged to include written comments on each item to justify and/or qualify their agreements or disagreements. Following each round, the panelists are provided with summaries of the votes of other panelists, comments of other panelists, and a reminder of their own vote on each item. The panelists are given a chance to change their votes in view of the votes and comments of other anonymous panelists.

### 2.2. Key Contact Experts

Before conducting the Delphi iterative rounds, we contacted and invited 4 oncologists, 3 exercise and medicine specialists, and 2 researchers who conducted research on the impact of exercise on cancer, 2 cancer patients, and 3 survivors (Figure 1). The patients and survivors had academic degrees in biology or one of the medical fields. We used key contacts in the field to identify the interviewees. The participants were interviewed on their opinions and views of what knowledge items primary healthcare providers and CAM practitioners need to know on the impact of exercise in stimulating anticancer immunity and suppressing cancerous cells. The interviewees were asked to list knowledge items they believed primary healthcare providers and CAM practitioners caring for cancer patients and survivors should know. We recorded the knowledge items mentioned by the interviewees.

### 2.3. Literature Search and Review

To complement and expand the list of knowledge items provided by the key contact experts, we conducted an extensive literature review to search for and identify all potential effects of exercise on stimulating anticancer immunity and suppressing cancerous cells [19, 22, 23, 27–29, 43–51]. The aim of this step was to identify all potential effects of exercise on stimulating anticancer immunity and suppressing cancerous cells that were reported in the literature. Potential effects of exercise on stimulating anticancer immunity and suppression of cancerous cells were collected from studies reported in the literature (Figure 1). In line with the hierarchy of scientific evidence, meta-analyses and systematic reviews of randomized clinical trials provided the strongest evidence. The hierarchy of scientific evidence categorizes the quality of evidence in descending order from the strongest to the weakest as follows: meta-analysis and systematic reviews of randomized controlled clinical trials, systematic reviews of randomized clinical trials, randomized controlled clinical trials, reviews of quasirandomized and nonrandomized clinical trials, quasirandomized and nonrandomized clinical trials, cohort studies, case-control studies, cross-sectional studies, reviews of animal and* in vitro* mechanistic studies, animal studies,* in vitro* mechanistic studies, and opinion papers. Potential effects of exercise on cancer and its therapy retrieved from the literature with their sources are summarized in Supplementary Table S1.

### 2.4. Ratings and Comments from Researchers and Experts in the Field

We then identified 20 researchers and experts who conducted research and published articles on exercise and cancer (Figure 1). The potential experts were identified using the advanced search option on PubMed database. We used and combined MeSH terms like “cancer” AND “exercise”, AND “immunity”. Articles that were judged relevant to the topic being investigated were manually searched [38]. The corresponding author was identified and emailed with an invitation providing the background and the objectives of the study and the list of knowledge items either provided by the interviewees or found in the literature. The aim of this step was to obtain feedback from experts in the field and enrich the list of the knowledge items to be used later in the iterative Delphi rounds. The corresponding author could also refer or forward the invitation to one of the authors on the article to respond. The researchers and experts who agreed to participate were asked to rate each item in the list and suggest further items to be included.

### 2.5. Drafting and Piloting

All knowledge items provided by the interviewees during the interviews, found in the literature, and those suggested by the researchers and experts were phrased by the authors into statements. The statements were compiled into a questionnaire. The questionnaire was piloted for clarity and understanding with 5 primary healthcare providers and 3 CAM practitioners (Figure 1). The primary healthcare providers and CAM practitioners who participated in the pilot study were identified by contacts in the field. They had more than 5 years of practice experience, prior knowledge of the effects of exercise on cancer immunity and suppression of cancerous cells, and were actively involved in providing healthcare for cancer patients and survivors. Participants were asked to rate each statement for clarity and comprehensibility. Under each statement, the participants were provided with a space to suggest rephrasing the statement in case needed. The participants suggested that some statements could be divided into two separate statements like “evidence from various epidemiological studies demonstrated that habitual exercise reduced the risks of at least 13 types of cancers and reduced recurrence of colon, prostate, and breast cancer”. This phrase was divided into two separate statements as “evidence from various epidemiological studies demonstrated that habitual exercise reduced the risks of at least 13 types of cancers” and “evidence from various epidemiological studies demonstrated that habitual exercise reduced recurrence of colon, prostate, and breast cancer”.

### 2.6. Panel of Healthcare Providers and CAM Practitioners

In this study, we used a purposive sampling technique to identify, invite, and recruit panelists to compose a panel of healthcare providers and CAM practitioners (Figure 1). We used key contacts in the field to identify, invite, and recruit the panelists [32, 38–42]. Recruitment of the panelists ensured that they possessed knowledge of the topic being investigated. The panelists were selected based on their academic qualifications, length of experience in the field, and number of cancer patients or survivors they interacted with. The panelists were approached and invited to take part in the study based on the following inclusion criteria: (1) licensed to practice medicine or CAM, (2) having at least 5 years of practicing experience in the field, (3) having prior knowledge of the effects of exercise on immunity and cancer, (4) interaction with more than 5 cancer patients or survivors, and (5) providing consent to participate in the study. The design of the study and its objectives were explained to the potential participants.

### 2.7. Voting and Commenting in the Iterative Delphi Rounds

#### 2.7.1. The First Delphi Round

The panelists received questionnaires that contained 3 sections. In the first section, the panelists were requested to include their practice and sociodemographic variables like gender, age, academic degree/specialty, employer, number of years in practice, and approximate number of cancer patients or survivors interacted with per month.

The second section contained 4 questions to explore the views and opinions of the panelists on educating/training primary healthcare providers and CAM practitioners on the role of exercise in stimulating anticancer immunity and suppressing cancerous cells. The panelists were asked (1) if they agreed that there was lack of training/education about the potential roles of exercise in cancer prevention and therapy in the curricula of primary healthcare providers and CAM practitioners, (2) if they thought that there should be more efforts to increase the knowledge of primary health providers and CAM practitioners on how exercise can stimulate anticancer immunity and suppress cancerous cells, and (3) if they though that such training/education might improve healthcare delivery and promote the wellbeing of cancer patients and survivors.

The third section contained a list of 49 knowledge items in the form of statements and the panelists were requested to express the level of their disagreement or agreement on a Likert-scale of 1-9. Voting 1-3 indicated that the panelist disagreed with the statement. This meant that the panelist was of the opinion that the knowledge item was not important and it should not be included in the core list of knowledge items to be considered by educators, trainer, and/or regulatory bodies when designing training/educational intervention and/or continuing education to enrich knowledge and expand understanding of primary healthcare providers and CAM practitioners on how exercise might stimulate anticancer immunity and suppress cancerous cells. Voting 7-9 indicated that the panelist agrees with the statement. This meant that the panelist was of the opinion that the knowledge item was important and it should be included in the core list of knowledge items to be considered by educators, trainer, and/or regulatory bodies when designing training/educational intervention and/or continuing education to enrich knowledge and expand understanding of primary healthcare providers and CAM practitioners on how exercise might stimulate anticancer immunity and suppress cancerous cells. The statement was considered equivocal when the panelists voted 4-6. This meant that the panelist was indecisive on the statement either to be considered as important or not. A space was left after each statement and the panelists were encouraged to include comments qualifying and/or justifying their votes.

*Analysis of the Votes.* For the analysis of votes, we used Microsoft Excel (Microsoft, 2013). Votes were entered into a spreadsheet and their descriptive statistics were generated. The first quartile (Q1), median (Q2), third quartile (Q3), and the interquartile range (IQR) were computed for each statement separately. The definitions of consensus were used as in previous studies in healthcare [32, 38–42]. Consensus was considered to have been achieved when the median score fell within the range of 7-9 and the IQR was between 0-2; the statement was included in the core list of important knowledge items. Consensus was said to have been achieved when the median score fell within the range 1-3 and the IQR was between 0-2, the statement was excluded from the core list of important knowledge items. The statement was considered equivocal and was subjected to a second Delphi round when either the median score was in the range 4-6 or the IQR was more than 2.

#### 2.7.2. The Second Delphi Round

Statements that were considered equivocal in the first Delphi round were included into a revised questionnaire and were subjected to a second Delphi round. As in previous studies, we provided the panelists with summaries of the comments made by other anonymous panelists on each comment, median and IQR of the votes on each statement, and a reminder of the panelist's own votes. The panelists were given a chance to change their votes in view of the comments and votes of the other panelists. The votes of the second Delphi round were analyzed using the same definitions of consensus as in the first Delphi round.

### 2.8. Ethical Approval

This study was approved by the Institutional Review Board (IRB) of An-Najah National University. The panelists provided consent before taking part in the study knowing that the Delphi technique was a semianonymous method. This meant that the identity of the panelists was known to the investigator but not to the other panelists. Votes and comments weighed equally in the analysis.

## 3. Results

### 3.1. Researcher and Expert Key Contacts

A total of 10 out of the 20 researchers and experts who were emailed responded and consented to participate in the study. A total of 3 reminder emails were sent to those who did not respond. The questionnaire was returned by 9 researchers and experts giving a response rate of 45% of those initially invited to take part. The participants were of both genders, belonged to different age groups, from different backgrounds, and with variable extensive experience in exercise and cancer. The participants were from the US, UK, Germany, France, Norway, and Palestine. The researchers and experts rated each item and suggested adding additional items. Researchers and experts suggested adding items related to the molecular mechanisms to enrich knowledge and expand the understanding of primary healthcare providers and CAM practitioners on how exercise related physiologic and endocrine changes might impact cancer. Researchers and experts suggested modifying some items for clarity and promoting understanding. The items were revised accordingly and included in the questionnaire for the Delphi rounds.

### 3.2. The Delphi Rounds among the Panel of Participants

#### 3.2.1. Response Rate

Responses were obtained from all the 65 healthcare providers and CAM practitioner panelists in the first Delphi round, giving a response rate of 100%. However, in the second Delphi round, responses were obtained from 58 panelists giving a response rate of 89.2%.

#### 3.2.2. Practice and Sociodemographic Variables of the Panelists

The median age of the panelists was 52 with an IQR of 15 years. Of all panelists, 47 (72.3%) were 45 years old and above. More than half (56.9%) of the panelists were male in gender. The majority (73.9%) of the panelists who took part in this study were either primary healthcare or CAM providers. The majority (61.5%) of the participants were employed in primary healthcare facilities (governmental or private). The majority (83.1%) of the panelists were in practice for more than 10 years. More than half (56.9%) of the panelists interacted with 5-9 patients with or survivors of cancer per month. The detailed practice and sociodemographic variables of the panelists who took part in this study are shown in Table 1.

#### 3.2.3. Views and Opinions of the Panelists on Educating/Training Primary Healthcare Providers and CAM Practitioners on the Role of Exercise in Stimulating Anticancer Immunity

When surveyed for their views and opinions, the vast majority (96.6%) of the panelists who took part in this study agreed with the literature that there was a lack of training/education with regard to the potential roles of exercises in stimulating anticancer immunity. Similarly, the vast majority (98.5%) of the panelists were of the opinion that more efforts were needed to increase knowledge of primary health providers and CAM practitioners on how exercise can stimulate anticancer immunity and suppress cancerous cells. The majority (78.5%) of the panelists believed that training/educating primary healthcare providers and CAM practitioners on the roles of exercise would improve healthcare delivery and promote the wellbeing of cancer patients and survivors. The detailed responses of the panelists are shown in Table 2.

#### 3.2.4. Knowledge Items on Which Consensus Was Achieved to Be Considered by Educators, Trainers, and/or Health Regulatory Bodies for Designing Training/Educational Course or Continuing Education for Primary Healthcare Providers and CAM Practitioners on the Roles of Exercise in Stimulating Anticancer Immunity

Of the 49 items initially presented to the panelists, consensus was achieved on 29 (59.2%) items in the first Delphi round. Items on which consensus was not achieved in the first Delphi round were included into a revised questionnaire and were subjected to a second iterative Delphi round. In the second Delphi round, consensus was achieved on further 16 (32.7%) items. In total, consensus was achieved on 45 items in 6 categories. Of those, 9 (20.0%) were general items on recommending moderate to vigorous habitual exercise, 16 (35.6%) were related to the effects of habitual exercise on the functions of immune system and exposure to carcinogens, 12 (26.7%) were related to the effects of habitual exercise on anticancer therapies, 3 (6.7%) were related to the effects of habitual exercise on metastasis of cancer, 3 (6.7%) were related to the effects of habitual exercise on metabolism within tumors, and 2 (4.4%) were related to the role of myokines release induced by habitual exercise. Details of these items are shown in Table 3. The panelists were of the opinion that these items were important and should be considered by educators, trainers, and/or regulatory bodies when designing training/educational intervention and/or continuing education to enrich knowledge and expand understanding of primary healthcare providers and CAM practitioners on how exercise might stimulate anticancer immunity and suppress cancerous cells.

#### 3.2.5. Items on Which Consensus Was Not Achieved Following the Two Iterative Delphi Rounds

Following the two iterative Delphi rounds, consensus was not achieved on 4 (8.2%) of the 49 items presented to the panelists. These items remained equivocal, i.e., might be considered by educators, trainers, and/or regulatory bodies when designing training/educational intervention and/or continuing education to enrich knowledge and expand understanding of primary healthcare providers and CAM practitioners on how exercise might stimulate anticancer immunity and suppress cancerous cells or not depending on the individual needs of the trainers/educators, and/or regulatory bodies. These equivocal items are shown in Table 4.

## 4. Discussion

In the present study, we sought for the first time formal consensus on important knowledge items that primary healthcare providers and CAM practitioners need to know on how exercise might stimulate anticancer immunity and suppress cancerous cells. Little guidance exists on what information and knowledge items should be included and discussed during a training/educational and/or continuing educational intervention designed to enrich knowledge and expand understanding of primary healthcare providers and CAM practitioners on how exercise might stimulate anticancer immunity and suppress cancerous cells [49]. This consensual core list of important knowledge items could be considered by trainers, educators, and/or regulatory bodies at the time of designing training/educational intervention and/or continuing education for primary healthcare providers and CAM practitioners. Therefore, the benefits of such consensual core list are multifold and including (1) guiding educators, trainers, and/or regulatory bodies to develop educational and/or training interventions to educate and/or train primary healthcare providers and CAM practitioners on how exercise and might stimulate anticancer immunity and suppress cancerous cells, (2) enhancing survivorship of cancer patients and improve their quality of life, (3) expanding the role that primary healthcare providers and CAM practitioners in providing better care for cancer patients and survivors, and (4) promoting congruence in healthcare delivery and shared decision making in cancer healthcare systems.

In the present study, 45% of the researcher and experts contacted to rate and comment on the knowledge items responded and returned the questionnaire. Additionally, all invited healthcare providers and CAM practitioners responded and returned the questionnaire in the first Delphi round. In the second Delphi round, the vast majority of the panelists retuned the revised questionnaire. These high response rates add to the validity and strength of the present study. Although the sample size was comparatively small, researchers and experts who rated the knowledge items and commented on the knowledge items were of different backgrounds, had extensive experience in conducting research on the effects of exercise on stimulating anticancer immunity and suppressing cancerous cells, and had experience in caring for cancer patients and survivors. Moreover, the healthcare providers and CAM practitioners who participated in the Delphi rounds were of both genders, belonged to different age groups, were employed by the governmental and private sectors, had different degrees and specialties, and, more importantly, interacted with a considerable number of cancer patients and survivors over an extended years of experience in the field. Such diversity might add validity, strength, and suitability of considering the knowledge items that the panelists finally agreed upon by the end of the Delphi rounds by trainers, educators, and/or regulatory bodies at the time of designing training/educational intervention and/or continuing education for primary healthcare providers and CAM practitioners on the role of exercise in stimulating anticancer immunity and suppressing cancerous cells.

In this study, the panelists agreed that the cancer curricula of primary healthcare providers and CAM practitioners were deficient. Furthermore, the panelists agreed that more efforts were needed to increase knowledge of primary healthcare providers on how exercise might stimulate anticancer immunity and suppress cancerous cells. These finding were consistent with findings of other studies conducted elsewhere in the world [33–37]. These studies have shown that cancer education in the medical schools, training, professional development, and continuing education was underdeveloped and needed improvements [33–35]. In Palestine, little attention was drawn on the effects of exercise on cancer and how exercise might stimulate anticancer immunity and suppress cancerous cells. Training sessions, worships, conferences, and scientific meetings can be great venues for disseminating latest research findings and sharing knowledge among participants [52, 53]. This study might highlight a need for future efforts to educate/train healthcare providers and CAM practitioners on the roles that exercise can play in cancer as currently clinical trials are being conducted to assess exercise as a therapeutic intervention in cancer [54].

Gold standards in considering what knowledge items trainers, educators, and/or regulatory bodies should consider at the time of designing training/educational intervention and/or continuing education for primary healthcare providers and CAM practitioners on the role of exercise in stimulating anticancer immunity and suppressing cancerous cells do not exist. Therefore, consensual core lists might serve as guidance for trainers, educators, and/or regulatory bodies while designing training/educational intervention and/or continuing education for primary healthcare providers and CAM practitioners on the role of exercise in stimulating anticancer immunity and suppressing cancerous cells. Using such consensual core lists might reduce bias, enhance transparency, impart validity, and add strength to judgmental methods while developing concepts [32]. Probably, consensual core lists appeal more to trainers, educators, and/or regulatory bodies than knowledge items improvised by educators/trainers.

Primary healthcare providers and CAM practitioners need to know all items presented in Table 3 in order to ensure knowledge enrichment and expansion of understanding of the role of exercise in stimulating anticancer immunity and suppressing cancerous cells. Through their relationships, healthcare providers are in a position to influence opinions, attitudes, beliefs, and practice of patients [55, 56]. Having knowledge of and ability to influence practices of patients, healthcare providers might make suitable recommendations concerning habitual exercise to stimulate anticancer immunity, suppress cancerous cells, and improve survival rates and wellbeing of cancer patients and survivors (Table 3). Knowing the effects of habitual exercise on metastasis of cancer and on the metabolic processes within tumors might enable healthcare providers recommend suitable exercise to patients and explain the anticipated benefits to their patients in easy and understandable way. Enriching knowledge and expanding understanding of the healthcare providers of mechanisms related to the role of exercise on the functioning of the immune system might enable them to keep pacing with the latest information on new therapeutic agents and trials involving cancer patients and survivors as well as recommending safe and promisingly effective exercise to reduce the risk of cancer or recurrence of cancer. Understanding the effects of exercise on anticancer therapy might help healthcare providers make suitable recommendations to increase the potency and efficacy of anticancer therapy, reduce the associated adverse effects, and improve the quality of life of patients with and survivors of cancer.

Although exercise is generally safe and was shown to significantly improve clinical, functional, and survival outcomes in cancer patients, it is noteworthy mentioning that cancer patients and survivors are vulnerable populations who are at increased risk of morbidity and mortality due to the significant consequences of cancer and its therapy. Therefore, healthcare providers and CAM practitioners should be aware that a one-size-fits-all approach would not be practically feasible for all patients; rather, recommendations for exercise should be personalized to meet the physical and psychosocial needs of the patients and survivors [5, 30]. While recommending exercises for cancer patients and survivors, healthcare providers and CAM practitioners need to take into consideration the following issues to balance their recommendations: (1) there are patient related factors like age and comorbidities that need to be taken into account while recommending suitable exercises for different patients who could be fragile to perform exercise, (2) the message should be formulated in a way that should not shame the patients that cancer was a consequence of their lifestyle choices, and (3) adherence to exercise is not the sole and/or vital factor that would determine the success of their treatment [30].

In this study, 4 knowledge items remained equivocal following the second Delphi round. These items might be considered or not depending on the preference of trainers/educators at the time of designing training/educational interventions to increase the knowledge of primary health providers and CAM partitions on how exercise might stimulate anticancer immunity and suppress cancerous cells.

The results of the present study might be interpreted after considering the following limitations. First, the survey could have been conducted in one of the conferences on exercise and cancer. Such conferences could have gathered a large number of experts in the field. Second, we did not include patients and or survivors of cancer in the two iterative Delphi rounds. However, inclusion of patients and survivors could have influenced agreements and disagreements on the different statements. Inclusion of patients and survivors was hampered by the nature of the statements explaining molecular mechanisms on how exercise might stimulate anticancer immunity and suppress cancerous cells. Finding cancer patients and survivors with prior sufficient knowledge on how exercise might stimulate anticancer immunity and suppress cancerous cells was difficult. Third, the sample size of the panel was not large. However, the sample size used in this study was in the range of those used in previous studies in healthcare [32, 38–42]. Finally, the sampling technique was purposive [57]. However, considering the nature of the study it was extremely difficult to use randomized sampling technique [32, 38–40]. Using purposive sampling was commonly practiced in previous studies in healthcare [32, 38–42].

## 5. Conclusion

In this study, formal consensus was achieved for the first time on a core list of knowledge items on how exercise might stimulate anticancer immunity and suppress cancerous cells. This core list might be considered at the time of developing training/educational interventions and/or continuous education for primary healthcare providers and CAM practitioners. Future studies are still needed to investigate if such consensual lists might improve congruence in cancer care continuum and improve survival rates and wellbeing of cancer patients and survivors.

## Figures and Tables

**Table 1:** Detailed practice and sociodemographic variables of the panelists who participated in the study (*n = 65*).

Variable	n	%
*Gender*		
Male	37	56.9
Female	28	43.1
*Age (in years)*		
< 45	18	27.7
≥ 45	47	72.3
*Academic degree/specialty*		
MD/PhD	7	10.8
MD (primary healthcare provider)	28	43.1
MD/public health	4	6.2
Oncologist	6	9.2
CAM provider	20	30.8
*Employer*		
Primary healthcare facility (government)	31	47.7
Primary healthcare facility (private)	9	13.8
Private practice	25	38.5
*Number of years in practice*		
5-9	11	16.9
10-14	23	35.4
15-19	18	27.7
≥ 20	13	20
*Approximate number of cancer patients or survivors seen per month*		
5-9	37	56.9
10-14	17	26.2
≥ 15	11	16.9

CAM: complementary and alternative medicine; MD: Doctor of Medicine; PhD: Doctor of Philosophy.

**Table 2:** Views and opinions of the panelists on educating/training primary healthcare providers and CAM practitioners on the role of exercise in stimulating anticancer immunity and suppressing cancerous cells.

#	Question	n	%
1	*Do you agree with the literature that there is lack of training/education about the potential roles of exercise in cancer prevention and therapy in the curricula of primary healthcare providers and CAM practitioners?*		
	Yes	63	96.9
	No	2	3.1
2	*Do you think that there should be more efforts to increase the knowledge of primary health providers and CAM practitioners on how exercise can stimulate anticancer immunity?*		
	Yes	64	98.5
	No	1	1.5
3	*Do you think that such training/education might improve healthcare delivery and promote the wellbeing of cancer patients and survivors?*		
	Yes	51	78.5
	No	14	21.5

**Table 3:** The core list of knowledge items on which consensus was achieved to be considered by educators, trainers, and/or health regulatory bodies for designing training/educational course or continuing education for primary healthcare providers and CAM practitioners on the roles of exercise in stimulating anticancer immunity and suppressing cancerous cells.

#	Item	Delphi round on which consensus was achieved
	*Recommending exercise to prevent cancer and improve survival rates and wellbeing of cancer patients and survivors*	
1	Sedentary lifestyle was observed to be associated with several common types of cancer.	2
2	Evidence from various epidemiological studies demonstrated that exercise reduced the risks of at least 13 types of cancer including endometrial, colorectal, breast, and lung cancers.	2
3	Evidence from various epidemiological studies demonstrated that exercise reduced recurrence of colon, prostate, and breast cancer.	1
4	A growing body of research has demonstrated that exercise improved survival in patients who have developed certain types of cancer.	2
5	Evidence from various animal studies demonstrated that exercise reduced incidence, growth, and metastasis of tumors.	1
6	A growing body of research has demonstrated that exercise improved objective physiologic measures related to physical function, body composition, and cardiopulmonary fitness of cancer patients and survivors.	1
7	A growing body of research has demonstrated that exercise improved reported outcomes related to sleep, fatigue, life satisfaction, and quality of life of cancer patients and survivors.	1
8	Cancer care providers should know that engaging cancer patients and survivors in exercise might promote their adherence to health life style like healthy diet and quitting smoking.	1
9	Evidence from various cancer animal models demonstrated that exercise controlled progression of cancer, regulated tumor growth, alleviated side effects of treatment, and improved therapy outcomes.	1

	*The effects of exercise on metastasis of cancer*	
1	Cancer care providers should know that exercise has the potential to reduce the rate of tumor growth.	1
2	Cancer care providers should know that exercise induced molecular factors that might be capable of interfering with tumor formation.	1
3	Cancer care providers should know that exercise stimulated the release of catecholamines that activate the Hippo and YAP signaling pathway which is implicated in tumor formation.	2

	*The effects of exercise on metabolism within tumors*	
1	Cancer care providers should know that tumors favored aerobic glycolysis to support high energy demands within rapidly proliferative environments of the tumor.	1
2	Cancer care providers should know that tumors were susceptible to increased energy stress during exercise.	1
3	Cancer care providers should know that exercise regulated metabolism within tumors probably through inhibiting the phosphatidylinositol-3-kinase (PI3K)/protein kinase B (PKB (Akt))/mammalian target of rapamycin (mTOR) (PI3K-Akt-mTOR) signaling pathway.	2

	*The effects of exercise on the functions of immune system and exposure to carcinogens*	
1	Cancer care providers should know that exercise increased the number of natural killer cell and their cytotoxic activity.	1
2	Cancer care providers should know that exercise increased monocytes and macrophages in number and function. This included increasing their antitumor cytotoxic activity and their ability to produce cytokines that suppressed cancerous cells.	1
3	Cancer care providers should know that exercise decreased the number and function of proinflammatory monocytes and proinflammatory cytokines.	1
4	Cancer care providers should know that exercise enhanced T-cell priming and antigen presenting by increasing expression of dendritic cells, interleukin (IL-4), and interferon (IFN-*γ*) expressing T-cells.	1
5	Cancer care providers should know that exercise improved adaptive immunity by increasing the number of naïve CD8+ T-cells, decreasing the number of senescent/exhausted CD4^+^ and CD8^+^ T-cells.	2
6	Cancer care providers should know that exercise mobilized and redistributed cytotoxic immune cells.	1
7	Cancer care providers should know that exercise increased the levels of chemokines attracting immune cells, natural killer cell-activating receptor ligands, and ligands that reduce blockade check-points of immune cells.	1
8	Cancer care providers should know that exercise increased the number of neutrophils and their production of antitumor peroxides and free radicals.	1
9	Cancer care providers should know that exercise increased interferon levels and cytotoxic natural killer and T-cells infiltration of tumors.	1
10	Cancer care providers should know that exercise decreased levels of lactate resulted from high aerobic glycolysis and thus, reduced suppressive effects of lactate on the functions of cytotoxic immune cells like T-cells.	2
11	Cancer care providers should know that exercise increased mobilization of cytotoxic immune cells through different mechanisms that involved shear stress induced by blood flow and adrenergic signaling. These immobilized cytotoxic immune cells might identify and eradicate cancerous cells.	2
12	Cancer care providers should know that habitual exercise might induce hyperthermia which can regulate and delay growth of tumors and increase infiltration of tumors by natural killer cells by increasing the diameter of blood vessels within the tumor.	1
13	Cancer care providers should know that exercise increased body temperature which in turn induced interleukin (IL-6) trans-signaling and subsequently made blood vessels within the tumor more permissible to cytotoxic cells.	1
14	Cancer care providers should know that exercise speeded up the passage of food through the large intestine and thus reduced exposure of the colon to carcinogens.	1
15	Cancer care providers should know that exercise altered fecal pH and modified the intestinal flora and thus reduced formation of carcinogens.	1
16	Cancer care providers should know that exercise might reduce the conversion of steroids to more potent carcinogens.	1

	*The role of myokines release induced by exercise *	
1	Cancer care providers should know that exercise stimulated skeletal muscles to release myokines. Released myokines like Oncostatin M, Irisin, and SPARC have the potential to inhibit cancer cells *in vitro*.	2
2	Cancer care providers should know that myokines released during exercise stimulated the release of cytokines, which in turn induced the release of interleukins. Interleukins (for example IL-6) were known to promote proliferation, differentiation, and maturation of natural killer and T-cells.	2

	*The effects of exercise on anticancer therapies*	
1	Cancer care providers should know that exercise had the potential to reduce tumor-induced muscle mass loss.	2
2	Cancer care providers should know that exercise might help reduce intramuscular protein degradation associated with chemotherapeutic agents.	2
3	Cancer care providers should know that exercise might induce the hormone ghrelin which induced appetite and reduced anorexia.	2
4	Cancer care providers should know that exercise had the potential to stimulate the release of anti-inflammatory cytokines and reduce the levels of proinflammatory factors in cancer states.	1
5	Cancer care providers should know that exercise have the potential to reduce body fats and cardiovascular risk factors in cancer states.	1
6	Cancer care providers should know that exercise had the potential to reduce the symptoms of anxiety, depression, and cognitive problems associated with cancer itself and anticancer therapies. Symptoms of depression were seen when kynurenine, which is a metabolite of tryptophan, crossed the blood-brain barrier. During exercise, PGC-1*α* transcription factor was upregulated which subsequently increased metabolism of kynurenine into kynurenic acid that cannot cross the blood-brain barrier.	2
7	Cancer care providers should know that exercise improved muscle strength which was a powerful predictor of patient survival after surgery for cancers.	1
8	Cancer care providers should know that exercise had the potential to improve the potency and efficacy of anticancer drugs.	2
9	Cancer care providers should know that exercise had the potential to reduce the toxicity of anticancer drugs.	2
10	Cancer care providers should know that exercise improved blood flow; this might improve delivery of adequate concentrations of anticancer agents to tumors.	1
11	Cancer care providers should know that exercise improved recovery and reduced postoperative complications in patients undergoing surgery for solid tumors.	1
12	Cancer care providers should know that exercise could protect patients with and survivors of cancer from comorbidities.	1

**Table 4:** Items on which consensus was not achieved following the two iterative Delphi rounds.

		First Delphi round		Second Delphi round	
#	Item	Median	IQR	Median	IQR
1	Cancer care providers should know that exercise interventions were unable to eradicate or significantly reduce already established tumors.	5	3	6	3
2	Cancer care providers should know that exercise might deplete reserves in the muscles and amino acids in the plasma. This might limit supply of energy to the immune cells.	4	2	4	3
3	Cancer care providers should know that exercise might increase production of free radicals, at least for short periods of time. This might promote tumors in genetically predisposed individuals.	4	4	5	4
4	Cancer care providers should know that exercise increased the need of antioxidant supplements.	6	3	7	3

IQR: interquartile range.

## Data Availability

Data supporting the results reported in this published article can be found in the Results Section and as Supplementary Materials with this manuscript or by contacting the corresponding author.

## Supplementary Materials

Supplementary Materials Supplementary Table S1 provides potential effects of exercise on cancer and its therapy retrieved from the literature.
