# Supplementary material for: What Do Primary Healthcare Providers and Complementary and Alternative Medicine Practitioners in Palestine Need to Know about Exercise for Cancer Patients and Survivors: A Consensual Study Using the Delphi Technique
Source: Evid Based Complement Alternat Med. 2019 Apr 17;2019:7695818. doi: 10.1155/2019/7695818 (PMC6500610; doi:10.1155/2019/7695818)
Supplement: Supplementary Materials — Supplementary Table S1 provides potential effects of exercise on cancer and its therapy retrieved from the literature. [file 7695818.f1.docx]

**Supplementary Materials**

**Supplementary Table S1:** Summary of potential effects of exercise on cancer and its therapy retrieved from the literature

| **Knowledge item** | **Evidence** | **Reference** |
| --- | --- | --- |
| **Effects of exercise on risk of developing cancer, survival rates, and wellbeing of patients and survivors** |  |  |
| Prolonged sedentary behavior increases the risk of several common types of cancers including endometrial, colorectal, breast, and lung cancers. | Meta-analysis of prospective studies conducted in humans | [[1](#_ENREF_1)] |
| Physical activity reduces risk of certain types of cancer. | Meta-analysis and systematic review of studies conducted in humans | [[2](#_ENREF_2)] |
| Exercise reduces recurrence of certain types of cancer. | Systematic review of epidemiological and randomized controlled trials conducted in humans | [[3](#_ENREF_3)] |
| Exercise improves survival rates among patients with certain types of cancers. | Meta-analysis and systematic review of studies conducted in humans | [[4](#_ENREF_4)] |
| Exercise reduces tumor size, growth, and metastasis in animal models. | Meta-analysis and systematic review of preclinical data conducted in animal models, notably rodents | [[5](#_ENREF_5), [6](#_ENREF_6)] |
| Exercise has positive effects on objective physiologic measures related to physical function, body composition, and cardio-pulmonary fitness of cancer patients and survivors. | Meta-analysis and systematic reviews of studies conducted in humans | [[7-11](#_ENREF_7)] |
| Engaging cancer patients and survivors in exercise might promote their adherence to healthy lifestyle including healthy diet and physical activity. | Meta-analysis and systematic reviews of studies conducted in humans, an integrative data analysis, and review of studies conducted in humans | [[12-14](#_ENREF_12)] |
| **The effects of exercise on metastasis of cancer** |  |  |
| Exercise improves the systemic pro-inflammatory profile in cancer patients and survivors and might consequently improve the immunological responses. | Meta-analysis and systematic reviews of studies conducted in humans, animal models, and *in vitro* mechanistic studies | [[5](#_ENREF_5), [15-19](#_ENREF_15)] |
| Exercise induces molecular factors that might be capable of interfering with tumor formation. | Reviews of animal and *in vitro* mechanistic studies | [[18](#_ENREF_18), [20](#_ENREF_20)] |
| Exercise stimulates the release of catecholamines that activate the Hippo and YAP signaling pathway which is implicated in tumor formation. | Mechanistic studies using human exercise–conditioned serum from patients and other *in vitro* studies | [[18](#_ENREF_18), [21-23](#_ENREF_21)] |
| **The effects of habitual exercise on metabolism within tumors** |  |  |
| Tumors favor aerobic glycolysis to support high energy demands within a rapidly proliferative environments of the tumor. Exercise might target the Warburg-type highly glycolytic metabolism within tumor cells and inhibit glycolysis. | Review of studies conducted in animal models | [[24-26](#_ENREF_24)] |
| Tumors are susceptible to increased energy stress during habitual exercise. | Reviews of animal and *in vitro* mechanistic studies | [[24-26](#_ENREF_24)] |
| Exercise regulates metabolism within tumors probably through inhibiting the phosphatidylinositol-3-kinase (PI3K)/protein kinase B (PKB (Akt))/mammalian target of rapamycin (mTOR) (PI3K-Akt-mTOR) signaling pathway. | Reviews of *in vitro* mechanistic studies | [[22](#_ENREF_22)] |
| **The effects of exercise on the functions of immune system and exposure to carcinogens** |  |  |
| Exercise increases the number of natural killer cell and their cytotoxic activity. | Systematic reviews of studies in humans and mechanistic *in vitro* studies | [[27-32](#_ENREF_27)] |
| Exercise increases monocytes and macrophages in number and function. This include increasing their anti-tumor cytotoxic activity and their ability to produce cytokines that suppress cancerous cells. | Systematic reviews of studies conducted in humans, animals, and other *in vitro* mechanistic studies | [[5](#_ENREF_5), [31](#_ENREF_31), [33-36](#_ENREF_33)] |
| Exercise decreases the number and function of pro-inflammatory monocytes and pro-inflammatory cytokines. | Systematic and narrative reviews of studies conducted in humans, animals, and other *in vitro* studies | [[20](#_ENREF_20), [31](#_ENREF_31), [34](#_ENREF_34), [37-42](#_ENREF_37)] |
| Exercise enhances T-cell priming and antigen presenting by increasing expression of dendritic cells, IL-4, and IFN-γ expressing T-cells. | Systematic reviews of studies conducted in humans, animals, and other *in vitro* studies | [[10](#_ENREF_10), [25](#_ENREF_25), [43-47](#_ENREF_43)] |
| Exercise improves adaptive immunity by increasing the number of naïve CD8+ T-cells, decreasing the number of senescent/exhausted CD4^+^ and CD8^+^ T-cells. | Systematic reviews of studies conducted in humans, animals, and other *in vitro* studies | [[34](#_ENREF_34), [43](#_ENREF_43), [47-50](#_ENREF_47)] |
| Exercise mobilizes and redistributes cytotoxic immune cells. | Reviews of studies conducted in humans, animals, and other *in vitro* studies | [[5](#_ENREF_5), [50-53](#_ENREF_50)] |
| Exercise increases the levels of chemokines attracting immune cells, natural killer cell-activating receptor ligands, and ligands that reduce blockade check-points of immune cells. | Reviews of studies conducted in humans, animals, and other *in vitro* studies | [[34](#_ENREF_34), [54-58](#_ENREF_54)] |
| Exercise increases the number of neutrophils and their production of antitumor peroxides and free radicals. | Reviews of studies conducted in humans, animals, and other *in vitro* studies | [[5](#_ENREF_5), [33](#_ENREF_33), [59](#_ENREF_59), [60](#_ENREF_60)] |
| Exercise increase interferon levels and cytotoxic NK and T cells infiltration of tumors. | Reviews of studies conducted in humans, animals, and other *in vitro* studies | [[10](#_ENREF_10), [27](#_ENREF_27), [47](#_ENREF_47), [61-63](#_ENREF_61)] |
| Exercise decreases levels of lactate resulted from high aerobic glycolysis and thus, reduce suppressive effects of lactate on the functions of cytotoxic immune cells like T cells. | Reviews of studies conducted in humans, animals, and other *in vitro* studies | [[24](#_ENREF_24), [64-66](#_ENREF_64)] |
| Exercise increases mobilization of cytotoxic immune cells through different mechanisms that involve shear stress induced by blood flow and adrenergic signaling. These immobilized cytotoxic immune cells might identify and eradicate cancerous cells. | Reviews of studies conducted in humans, animals, and other *in vitro* studies | [[18](#_ENREF_18), [27](#_ENREF_27), [67](#_ENREF_67), [68](#_ENREF_68)] |
| Exercise might induce hyperthermia which can regulate and delay growth of tumors and increase infiltration of tumors by natural killer cells by increasing the diameter of blood vessels within the tumor. | Meta-analysis and systematic reviews of studies in humans | [[46](#_ENREF_46), [69-71](#_ENREF_69)] |
| Exercise increases body temperature which in turn induces IL-6 trans-signaling and subsequently make blood vessels more permissible to cytotoxic T cells within the tumor. | Meta-analysis and systematic reviews of studies in humans | [[10](#_ENREF_10), [20](#_ENREF_20), [39](#_ENREF_39), [72](#_ENREF_72)] |
| There is an inverse association between exercise and colon cancer in men and women. | Meta-analysis and systematic reviews of studies in humans | [[73-76](#_ENREF_73)] |
| Exercise alters fecal pH and modify the intestinal flora, thus reduce formation of carcinogens. | Reviews of studies conducted in humans, animals, and other *in vitro* studies | [[76-79](#_ENREF_76)] |
| Exercise might reduce the conversion of steroids to more potent carcinogens. | Reviews of *in vitro* studies | [[24](#_ENREF_24), [80](#_ENREF_80)] |
| **The role of myokines release induced by habitual exercise** |  |  |
| Exercise stimulates skeletal muscles to release myokines. The released myokines like Oncostatin M, Irisin, and SPARC have the potential to inhibit cancer cells *in vitro*. | Reviews of *in vitro* studies | [[18](#_ENREF_18), [81](#_ENREF_81)] |
| Myokines released during habitual exercise stimulate the release of cytokines, which in turn, induce the release of interleukins. Interleukins (for example IL-6) are known to promote proliferation, differentiation, and maturation of natural killer and T cells. | Reviews of *in vitro* studies | [[18](#_ENREF_18), [20](#_ENREF_20), [82](#_ENREF_82), [83](#_ENREF_83)] |
| **The effects of habitual exercise on anticancer therapies** |  |  |
| Exercise have the potential to reduce tumor-induced muscle mass loss. | Reviews of studies conducted in humans | [[10](#_ENREF_10), [84](#_ENREF_84), [85](#_ENREF_85)] |
| Exercise might help reduce intramuscular protein degradation associated with chemotherapeutic agents. | Reviews of studies conducted in humans | [[24](#_ENREF_24), [86](#_ENREF_86)] |
| Exercise might induce the hormone ghrelin which induces appetite and reduces anorexia. | Reviews of studies conducted in humans and animal models | [[87-91](#_ENREF_87)] |
| Exercise have the potential to stimulate the release of anti-inflammatory cytokines and reduce the levels of pro-inflammatory factors in cancer states. | Systematic and narrative reviews of studies conducted in humans, animals, and other *in vitro* studies | [[20](#_ENREF_20), [31](#_ENREF_31), [34](#_ENREF_34), [37-42](#_ENREF_37)] |
| Exercise have the potential to reduce body fats and cardiovascular risk factors in cancer states. | Meta-analyses of clinical trials and systematic reviews | [[92-94](#_ENREF_92)] |
| Exercise has the potential to reduce the symptoms of anxiety, depression, and cognitive problems associated with cancer itself and anticancer therapies. Symptoms of depression were seen when kynurenine, which is a metabolite of tryptophan, crossed the blood-brain barrier. During exercise, PGC-1α transcription factor was upregulated which subsequently increased metabolism of kynurenine into kynurenic acid that cannot cross the blood-brain barrier. | Meta-analyses of randomized controlled trials and systematic reviews | [[9](#_ENREF_9), [67](#_ENREF_67), [95](#_ENREF_95), [96](#_ENREF_96)] |
| Exercise improves muscle strength which is powerful predictor of patient survival after surgery for cancers. | Reviews of studies conducted in humans and other mechanistic studies | [[18](#_ENREF_18), [81](#_ENREF_81)] |
| Exercise has the potential to improve the potency and efficacy of anticancer drugs. | Meta-analysis and systematic reviews of studies in humans | [[10](#_ENREF_10), [67](#_ENREF_67), [93](#_ENREF_93), [97](#_ENREF_97), [98](#_ENREF_98)] |
| Exercise has the potential to reduce the toxicity of anticancer drugs. | Meta-analysis and systematic reviews of studies in humans | [[93](#_ENREF_93), [99](#_ENREF_99), [100](#_ENREF_100)] |
| Exercise improves blood flow, this might improve delivery of adequate concentrations of anticancer agents to tumors. | Meta-analysis and systematic reviews of studies in humans | [[71](#_ENREF_71), [93](#_ENREF_93)] |
| Exercise improves recovery and reduce postoperative complications in patients undergoing surgery for solid tumors. | Meta-analysis and systematic reviews of studies in humans | [[101-103](#_ENREF_101)] |
| Exercise can protect patients with and survivors of cancer from co-morbidities. | Meta-analysis and systematic reviews of studies in humans | [[3](#_ENREF_3), [10](#_ENREF_10), [84](#_ENREF_84)] |

**References**

1. Shen D., Mao W., Liu T., et al. Sedentary behavior and incident cancer: a meta-analysis of prospective studies, *PloS one*. 2014; 9(8): e105709. doi: 10.1371/journal.pone.0105709.

2. Shaw E., Farris M. S., Stone C. R., et al. Effects of physical activity on colorectal cancer risk among family history and body mass index subgroups: a systematic review and meta-analysis, *BMC cancer*. 2018; 18(1): 71. doi: 10.1186/s12885-017-3970-5.

3. Cormie P., Zopf E. M., Zhang X., and Schmitz K. H. The Impact of Exercise on Cancer Mortality, Recurrence, and Treatment-Related Adverse Effects, *Epidemiologic reviews*. 2017; 39(1): 71-92. doi: 10.1093/epirev/mxx007.

4. McNeely M. L., Campbell K. L., Rowe B. H., Klassen T. P., Mackey J. R., and Courneya K. S. Effects of exercise on breast cancer patients and survivors: a systematic review and meta-analysis, *CMAJ : Canadian Medical Association journal = journal de l'Association medicale canadienne*. 2006; 175(1): 34-41. doi: 10.1503/cmaj.051073.

5. Eschke R. K., Lampit A., Schenk A., et al. Impact of Physical Exercise on Growth and Progression of Cancer in Rodents-A Systematic Review and Meta-Analysis, *Frontiers in oncology*. 2019; 935. doi: 10.3389/fonc.2019.00035.

6. Figueira A. C. C., Cortinhas A., Soares J. P., Leitao J. C., Ferreira R. P., and Duarte J. A. Efficacy of Exercise on Breast Cancer Outcomes: A Systematic Review and Meta-analysis of Preclinical Data, *International journal of sports medicine*. 2018; 39(5): 327-342. doi: 10.1055/s-0044-101149.

7. Mishra S. I., Scherer R. W., Snyder C., Geigle P. M., Berlanstein D. R., and Topaloglu O. Exercise interventions on health-related quality of life for people with cancer during active treatment, *Clinical otolaryngology : official journal of ENT-UK ; official journal of Netherlands Society for Oto-Rhino-Laryngology & Cervico-Facial Surgery*. 2012; 37(5): 390-392. doi: 10.1111/coa.12015.

8. Mishra S. I., Scherer R. W., Geigle P. M., et al. Exercise interventions on health-related quality of life for cancer survivors, *The Cochrane database of systematic reviews*. 2012; (8): CD007566. doi: 10.1002/14651858.CD007566.pub2.

9. Fong D. Y., Ho J. W., Hui B. P., et al. Physical activity for cancer survivors: meta-analysis of randomised controlled trials, *Bmj*. 2012; 344e70. doi: 10.1136/bmj.e70.

10. Stout N. L., Baima J., Swisher A. K., Winters-Stone K. M., and Welsh J. A Systematic Review of Exercise Systematic Reviews in the Cancer Literature (2005-2017), *PM & R : the journal of injury, function, and rehabilitation*. 2017; 9(9S2): S347-S384. doi: 10.1016/j.pmrj.2017.07.074.

11. Singh B., Spence R. R., Steele M. L., Sandler C. X., Peake J. M., and Hayes S. C. A Systematic Review and Meta-Analysis of the Safety, Feasibility, and Effect of Exercise in Women With Stage II+ Breast Cancer, *Archives of physical medicine and rehabilitation*. 2018; 99(12): 2621-2636. doi: 10.1016/j.apmr.2018.03.026.

12. Adams R. N., Mosher C. E., Blair C. K., Snyder D. C., Sloane R., and Demark-Wahnefried W. Cancer survivors' uptake and adherence in diet and exercise intervention trials: an integrative data analysis, *Cancer*. 2015; 121(1): 77-83. doi: 10.1002/cncr.28978.

13. Ormel H. L., van der Schoot G. G. F., Sluiter W. J., Jalving M., Gietema J. A., and Walenkamp A. M. E. Predictors of adherence to exercise interventions during and after cancer treatment: A systematic review, *Psycho-oncology*. 2018; 27(3): 713-724. doi: 10.1002/pon.4612.

14. Husebo A. M., Dyrstad S. M., Soreide J. A., and Bru E. Predicting exercise adherence in cancer patients and survivors: a systematic review and meta-analysis of motivational and behavioural factors, *Journal of clinical nursing*. 2013; 22(1-2): 4-21. doi: 10.1111/j.1365-2702.2012.04322.x.

15. Meneses-Echavez J. F., Ramirez-Velez R., Gonzalez-Jimenez E., Rio-Valle J. S., Perez M. J. S., and Montes E. M. Exercise training, inflammatory cytokines, and other markers of low-grade inflammation in breast cancer survivors: A systematic review and meta-analysis, *Journal of Clinical Oncology*. 2014; 32(26_suppl): 121-121. doi: 10.1200/jco.2014.32.26_suppl.121.

16. Jones L. W., Viglianti B. L., Tashjian J. A., et al. Effect of aerobic exercise on tumor physiology in an animal model of human breast cancer, *J Appl Physiol (1985)*. 2010; 108(2): 343-348. doi: 10.1152/japplphysiol.00424.2009.

17. Ashcraft K. A., Peace R. M., Betof A. S., Dewhirst M. W., and Jones L. W. Efficacy and Mechanisms of Aerobic Exercise on Cancer Initiation, Progression, and Metastasis: A Critical Systematic Review of In Vivo Preclinical Data, *Cancer research*. 2016; 76(14): 4032-4050. doi: 10.1158/0008-5472.CAN-16-0887.

18. Hojman P., Gehl J., Christensen J. F., and Pedersen B. K. Molecular Mechanisms Linking Exercise to Cancer Prevention and Treatment, *Cell metabolism*. 2018; 27(1): 10-21. doi: 10.1016/j.cmet.2017.09.015.

19. Hayes B. D., Brady L., Pollak M., and Finn S. P. Exercise and Prostate Cancer: Evidence and Proposed Mechanisms for Disease Modification, *Cancer epidemiology, biomarkers & prevention : a publication of the American Association for Cancer Research, cosponsored by the American Society of Preventive Oncology*. 2016; 25(9): 1281-1288. doi: 10.1158/1055-9965.EPI-16-0223.

20. Thomas R. J., Kenfield S. A., and Jimenez A. Exercise-induced biochemical changes and their potential influence on cancer: a scientific review, *British journal of sports medicine*. 2017; 51(8): 640-644. doi: 10.1136/bjsports-2016-096343.

21. Dethlefsen C., Hansen L. S., Lillelund C., et al. Exercise-Induced Catecholamines Activate the Hippo Tumor Suppressor Pathway to Reduce Risks of Breast Cancer Development, *Cancer research*. 2017; 77(18): 4894-4904. doi: 10.1158/0008-5472.CAN-16-3125.

22. Agostini D., Natalucci V., Baldelli G., et al. New Insights into the Role of Exercise in Inhibiting mTOR Signaling in Triple-Negative Breast Cancer, *Oxidative medicine and cellular longevity*. 2018; 20185896786. doi: 10.1155/2018/5896786.

23. Wei C., Wang Y., and Li X. The role of Hippo signal pathway in breast cancer metastasis, *OncoTargets and therapy*. 2018; 112185-2193. doi: 10.2147/OTT.S157058.

24. Hofmann P. Cancer and Exercise: Warburg Hypothesis, Tumour Metabolism and High-Intensity Anaerobic Exercise, *Sports*. 2018; 6(1). doi: 10.3390/sports6010010.

25. Ruiz-Casado A., Martin-Ruiz A., Perez L. M., Provencio M., Fiuza-Luces C., and Lucia A. Exercise and the Hallmarks of Cancer, *Trends in cancer*. 2017; 3(6): 423-441. doi: 10.1016/j.trecan.2017.04.007.

26. Gatenby R. A. and Gillies R. J. Why do cancers have high aerobic glycolysis?, *Nature Reviews Cancer*. 2004; 4891. doi: 10.1038/nrc1478.

27. Idorn M. and Hojman P. Exercise-Dependent Regulation of NK Cells in Cancer Protection, *Trends in molecular medicine*. 2016; 22(7): 565-577. doi: 10.1016/j.molmed.2016.05.007.

28. Shephard R. J. and Shek P. N. Effects of exercise and training on natural killer cell counts and cytolytic activity: a meta-analysis, *Sports medicine*. 1999; 28(3): 177-195. doi: 10.2165/00007256-199928030-00003.

29. Evans E. S., Hackney A. C., McMurray R. G., et al. Impact of Acute Intermittent Exercise on Natural Killer Cells in Breast Cancer Survivors, *Integrative cancer therapies*. 2015; 14(5): 436-445. doi: 10.1177/1534735415580681.

30. Zimmer P., Schenk A., Kieven M., et al. Exercise induced alterations in NK-cell cytotoxicity - methodological issues and future perspectives, *Exercise immunology review*. 2017; 2366-81. doi:

31. Kruijsen-Jaarsma M., Revesz D., Bierings M. B., Buffart L. M., and Takken T. Effects of exercise on immune function in patients with cancer: a systematic review, *Exercise immunology review*. 2013; 19120-143. doi:

32. Idorn M. and Thor Straten P. Exercise and cancer: from "healthy" to "therapeutic"?, *Cancer immunology, immunotherapy : CII*. 2017; 66(5): 667-671. doi: 10.1007/s00262-017-1985-z.

33. Fairey A. S., Courneya K. S., Field C. J., and Mackey J. R. Physical exercise and immune system function in cancer survivors: a comprehensive review and future directions, *Cancer*. 2002; 94(2): 539-551. doi: 10.1002/cncr.10244.

34. Schmidt T., van Mackelenbergh M., Wesch D., and Mundhenke C. Physical activity influences the immune system of breast cancer patients, *Journal of cancer research and therapeutics*. 2017; 13(3): 392-398. doi: 10.4103/0973-1482.150356.

35. Richards D. M., Hettinger J., and Feuerer M. Monocytes and macrophages in cancer: development and functions, *Cancer microenvironment : official journal of the International Cancer Microenvironment Society*. 2013; 6(2): 179-191. doi: 10.1007/s12307-012-0123-x.

36. Woods J. A. and Davis J. M. Exercise, monocyte/macrophage function, and cancer, *Medicine and science in sports and exercise*. 1994; 26(2): 147-156. doi:

37. Mills R. C., 3rd. Breast Cancer Survivors, Common Markers of Inflammation, and Exercise: A Narrative Review, *Breast cancer : basic and clinical research*. 2017; 111178223417743976. doi: 10.1177/1178223417743976.

38. LaVoy E. C., Fagundes C. P., and Dantzer R. Exercise, inflammation, and fatigue in cancer survivors, *Exercise immunology review*. 2016; 2282-93. doi:

39. Meneses-Echavez J. F., Correa-Bautista J. E., Gonzalez-Jimenez E., et al. The Effect of Exercise Training on Mediators of Inflammation in Breast Cancer Survivors: A Systematic Review with Meta-analysis, *Cancer epidemiology, biomarkers & prevention : a publication of the American Association for Cancer Research, cosponsored by the American Society of Preventive Oncology*. 2016; 25(7): 1009-1017. doi: 10.1158/1055-9965.EPI-15-1061.

40. Monteiro-Junior R. S., de Tarso Maciel-Pinheiro P., da Matta Mello Portugal E., et al. Effect of Exercise on Inflammatory Profile of Older Persons: Systematic Review and Meta-Analyses, *Journal of physical activity & health*. 2018; 15(1): 64-71. doi: 10.1123/jpah.2016-0735.

41. Goh J., Niksirat N., and Campbell K. L. Exercise training and immune crosstalk in breast cancer microenvironment: exploring the paradigms of exercise-induced immune modulation and exercise-induced myokines, *American journal of translational research*. 2014; 6(5): 422-438. doi:

42. Koelwyn G. J., Wennerberg E., Demaria S., and Jones L. W. Exercise in Regulation of Inflammation-Immune Axis Function in Cancer Initiation and Progression, *Oncology (Williston Park)*. 2015; 29(12): 908-920, 922. doi:

43. Turner J. E. and Brum P. C. Does Regular Exercise Counter T Cell Immunosenescence Reducing the Risk of Developing Cancer and Promoting Successful Treatment of Malignancies?, *Oxidative medicine and cellular longevity*. 2017; 20174234765. doi: 10.1155/2017/4234765.

44. Marconi R., Serafini A., Giovanetti A., et al. Cytokine Modulation in Breast Cancer Patients Undergoing Radiotherapy: A Revision of the Most Recent Studies, *International journal of molecular sciences*. 2019; 20(2). doi: 10.3390/ijms20020382.

45. Nilsson M. I., Bourgeois J. M., Nederveen J. P., et al. Lifelong aerobic exercise protects against inflammaging and cancer, *PloS one*. 2019; 14(1): e0210863. doi: 10.1371/journal.pone.0210863.

46. Buffart L. M., Sweegers M. G., May A. M., et al. Targeting Exercise Interventions to Patients With Cancer in Need: An Individual Patient Data Meta-Analysis, *Journal of the National Cancer Institute*. 2018; 110(11): 1190-1200. doi: 10.1093/jnci/djy161.

47. Sellami M., Gasmi M., Denham J., et al. Effects of Acute and Chronic Exercise on Immunological Parameters in the Elderly Aged: Can Physical Activity Counteract the Effects of Aging?, *Frontiers in immunology*. 2018; 92187. doi: 10.3389/fimmu.2018.02187.

48. Bigley A. B., Spielmann G., LaVoy E. C., and Simpson R. J. Can exercise-related improvements in immunity influence cancer prevention and prognosis in the elderly?, *Maturitas*. 2013; 76(1): 51-56. doi: 10.1016/j.maturitas.2013.06.010.

49. Witard O. C., Turner J. E., Jackman S. R., et al. High-intensity training reduces CD8+ T-cell redistribution in response to exercise, *Medicine and science in sports and exercise*. 2012; 44(9): 1689-1697. doi: 10.1249/MSS.0b013e318257d2db.

50. Campbell J. P. and Turner J. E. Debunking the Myth of Exercise-Induced Immune Suppression: Redefining the Impact of Exercise on Immunological Health Across the Lifespan, *Frontiers in immunology*. 2018; 9648. doi: 10.3389/fimmu.2018.00648.

51. Ali H. R., Provenzano E., Dawson S. J., et al. Association between CD8+ T-cell infiltration and breast cancer survival in 12,439 patients, *Annals of oncology : official journal of the European Society for Medical Oncology*. 2014; 25(8): 1536-1543. doi: 10.1093/annonc/mdu191.

52. Simpson R. J., Bigley A. B., Agha N., Hanley P. J., and Bollard C. M. Mobilizing Immune Cells With Exercise for Cancer Immunotherapy, *Exercise and sport sciences reviews*. 2017; 45(3): 163-172. doi: 10.1249/JES.0000000000000114.

53. Ingram C. and Visovsky C. Exercise intervention to modify physiologic risk factors in cancer survivors, *Seminars in oncology nursing*. 2007; 23(4): 275-284. doi: 10.1016/j.soncn.2007.08.005.

54. Argyle D. and Kitamura T. Targeting Macrophage-Recruiting Chemokines as a Novel Therapeutic Strategy to Prevent the Progression of Solid Tumors, *Frontiers in immunology*. 2018; 92629. doi: 10.3389/fimmu.2018.02629.

55. Susek K. H., Karvouni M., Alici E., and Lundqvist A. The Role of CXC Chemokine Receptors 1-4 on Immune Cells in the Tumor Microenvironment, *Frontiers in immunology*. 2018; 92159. doi: 10.3389/fimmu.2018.02159.

56. Vilgelm A. E. and Richmond A. Chemokines Modulate Immune Surveillance in Tumorigenesis, Metastasis, and Response to Immunotherapy, *Frontiers in immunology*. 2019; 10(333). doi: 10.3389/fimmu.2019.00333.

57. Idorn M. and Thor Straten P. Chemokine Receptors and Exercise to Tackle the Inadequacy of T Cell Homing to the Tumor Site, *Cells*. 2018; 7(8). doi: 10.3390/cells7080108.

58. Adraskela K., Veisaki E., Koutsilieris M., and Philippou A. Physical Exercise Positively Influences Breast Cancer Evolution, *Clinical breast cancer*. 2017; 17(6): 408-417. doi: 10.1016/j.clbc.2017.05.003.

59. Betof A. S., Dewhirst M. W., and Jones L. W. Effects and potential mechanisms of exercise training on cancer progression: a translational perspective, *Brain, behavior, and immunity*. 2013; 30 SupplS75-87. doi: 10.1016/j.bbi.2012.05.001.

60. Repka C. P. and Hayward R. Effects of an Exercise Intervention on Cancer-Related Fatigue and Its Relationship to Markers of Oxidative Stress, *Integrative cancer therapies*. 2018; 17(2): 503-510. doi: 10.1177/1534735418766402.

61. Kaya O. Effect of a four-week exercise program on the secretion of IFN-gamma, TNF-alpha, IL-2 and IL-6 cytokines in elite Taekwondo athletes, *Biomedical reports*. 2016; 5(3): 367-370. doi: 10.3892/br.2016.730.

62. Gleeson M. and Bishop N. C. The T cell and NK cell immune response to exercise, *Annals of transplantation*. 2005; 10(4): 43-48. doi:

63. Chamorro-Vina C., Valentin J., Fernandez L., et al. Influence of a Moderate-Intensity Exercise Program on Early NK Cell Immune Recovery in Pediatric Patients After Reduced-Intensity Hematopoietic Stem Cell Transplantation, *Integrative cancer therapies*. 2017; 16(4): 464-472. doi: 10.1177/1534735416679515.

64. Tosti K. P., Hackney A. C., Battaglini C. L., Evans E. S., and Groff D. Exercise in patients with breast cancer and healthy controls: energy substrate oxidation and blood lactate responses, *Integrative cancer therapies*. 2011; 10(1): 6-15. doi: 10.1177/1534735410387600.

65. Kessels E., Husson O., and van der Feltz-Cornelis C. M. The effect of exercise on cancer-related fatigue in cancer survivors: a systematic review and meta-analysis, *Neuropsychiatric disease and treatment*. 2018; 14479-494. doi: 10.2147/NDT.S150464.

66. Heywood R., McCarthy A. L., and Skinner T. L. Efficacy of Exercise Interventions in Patients With Advanced Cancer: A Systematic Review, *Archives of physical medicine and rehabilitation*. 2018; 99(12): 2595-2620. doi: 10.1016/j.apmr.2018.04.008.

67. Nakano J., Hashizume K., Fukushima T., et al. Effects of Aerobic and Resistance Exercises on Physical Symptoms in Cancer Patients: A Meta-analysis, *Integrative cancer therapies*. 2018; 17(4): 1048-1058. doi: 10.1177/1534735418807555.

68. Brown J. C., Rhim A. D., Manning S. L., et al. Effects of exercise on circulating tumor cells among patients with resected stage I-III colon cancer, *PloS one*. 2018; 13(10): e0204875. doi: 10.1371/journal.pone.0204875.

69. Nielsen P. J., Hafdahl A. R., Conn V. S., Lemaster J. W., and Brown S. A. Meta-analysis of the effect of exercise interventions on fitness outcomes among adults with type 1 and type 2 diabetes, *Diabetes research and clinical practice*. 2006; 74(2): 111-120. doi: 10.1016/j.diabres.2006.03.033.

70. Longo T. A., Gopalakrishna A., Tsivian M., et al. A systematic review of regional hyperthermia therapy in bladder cancer, *International journal of hyperthermia : the official journal of European Society for Hyperthermic Oncology, North American Hyperthermia Group*. 2016; 32(4): 381-389. doi: 10.3109/02656736.2016.1157903.

71. Beaudry R. I., Liang Y., Boyton S. T., et al. Meta-analysis of Exercise Training on Vascular Endothelial Function in Cancer Survivors, *Integrative cancer therapies*. 2018; 17(2): 192-199. doi: 10.1177/1534735418756193.

72. Rogers L. Q., Fogleman A., Trammell R., et al. Effects of a physical activity behavior change intervention on inflammation and related health outcomes in breast cancer survivors: pilot randomized trial, *Integrative cancer therapies*. 2013; 12(4): 323-335. doi: 10.1177/1534735412449687.

73. Wolin K. Y., Yan Y., Colditz G. A., and Lee I. M. Physical activity and colon cancer prevention: a meta-analysis, *British journal of cancer*. 2009; 100(4): 611-616. doi: 10.1038/sj.bjc.6604917.

74. Boyle T., Keegel T., Bull F., Heyworth J., and Fritschi L. Physical activity and risks of proximal and distal colon cancers: a systematic review and meta-analysis, *Journal of the National Cancer Institute*. 2012; 104(20): 1548-1561. doi: 10.1093/jnci/djs354.

75. Kyu H. H., Bachman V. F., Alexander L. T., et al. Physical activity and risk of breast cancer, colon cancer, diabetes, ischemic heart disease, and ischemic stroke events: systematic review and dose-response meta-analysis for the Global Burden of Disease Study 2013, *Bmj*. 2016; 354i3857. doi: 10.1136/bmj.i3857.

76. Quadrilatero J. and Hoffman-Goetz L. Physical activity and colon cancer. A systematic review of potential mechanisms, *The Journal of sports medicine and physical fitness*. 2003; 43(2): 121-138. doi:

77. Hibler E. Epigenetics and Colorectal Neoplasia: the Evidence for Physical Activity and Sedentary Behavior, *Current colorectal cancer reports*. 2015; 11(6): 388-396. doi: 10.1007/s11888-015-0296-z.

78. Cramer H., Lauche R., Klose P., Dobos G., and Langhorst J. A systematic review and meta-analysis of exercise interventions for colorectal cancer patients, *European journal of cancer care*. 2014; 23(1): 3-14. doi: 10.1111/ecc.12093.

79. Wolin K. Y., Yan Y., and Colditz G. A. Physical activity and risk of colon adenoma: a meta-analysis, *British journal of cancer*. 2011; 104(5): 882-885. doi: 10.1038/sj.bjc.6606045.

80. Westerlind K. C. Physical activity and cancer prevention--mechanisms, *Medicine and science in sports and exercise*. 2003; 35(11): 1834-1840. doi: 10.1249/01.MSS.0000093619.37805.B7.

81. Hojman P., Dethlefsen C., Brandt C., Hansen J., Pedersen L., and Pedersen B. K. Exercise-induced muscle-derived cytokines inhibit mammary cancer cell growth, *American journal of physiology Endocrinology and metabolism*. 2011; 301(3): E504-510. doi: 10.1152/ajpendo.00520.2010.

82. Mauer J., Denson J. L., and Bruning J. C. Versatile functions for IL-6 in metabolism and cancer, *Trends in immunology*. 2015; 36(2): 92-101. doi: 10.1016/j.it.2014.12.008.

83. Shalamzari S. A., Agha-Alinejad H., Alizadeh S., et al. The effect of exercise training on the level of tissue IL-6 and vascular endothelial growth factor in breast cancer bearing mice, *Iranian journal of basic medical sciences*. 2014; 17(4): 231-258. doi:

84. Kelley G. A. and Kelley K. S. Exercise and cancer-related fatigue in adults: a systematic review of previous systematic reviews with meta-analyses, *BMC cancer*. 2017; 17(1): 693. doi: 10.1186/s12885-017-3687-5.

85. Aversa Z., Costelli P., and Muscaritoli M. Cancer-induced muscle wasting: latest findings in prevention and treatment, *Therapeutic advances in medical oncology*. 2017; 9(5): 369-382. doi: 10.1177/1758834017698643.

86. Al-Majid S. and Waters H. The biological mechanisms of cancer-related skeletal muscle wasting: the role of progressive resistance exercise, *Biological research for nursing*. 2008; 10(1): 7-20. doi: 10.1177/1099800408317345.

87. Sever S., White D. L., and Garcia J. M. Is there an effect of ghrelin/ghrelin analogs on cancer? A systematic review, *Endocrine-related cancer*. 2016; 23(9): R393-409. doi: 10.1530/ERC-16-0130.

88. Mani B. K., Castorena C. M., Osborne-Lawrence S., et al. Ghrelin mediates exercise endurance and the feeding response post-exercise, *Molecular metabolism*. 2018; 9114-130. doi: 10.1016/j.molmet.2018.01.006.

89. Khatib M. N., Gaidhane A., Gaidhane S., and Quazi Z. S. Ghrelin as a Promising Therapeutic Option for Cancer Cachexia, *Cellular physiology and biochemistry : international journal of experimental cellular physiology, biochemistry, and pharmacology*. 2018; 48(5): 2172-2188. doi: 10.1159/000492559.

90. Khatib M. N., Shankar A. H., Kirubakaran R., et al. Ghrelin for the management of cachexia associated with cancer, *The Cochrane database of systematic reviews*. 2018; 2CD012229. doi: 10.1002/14651858.CD012229.pub2.

91. Nuri R., Moghaddasi M., Darvishi H., and Izadpanah A. Effect of aerobic exercise on leptin and ghrelin in patients with colorectal cancer, *Journal of cancer research and therapeutics*. 2016; 12(1): 169-174. doi: 10.4103/0973-1482.155982.

92. Fuller J. T., Hartland M. C., Maloney L. T., and Davison K. Therapeutic effects of aerobic and resistance exercises for cancer survivors: a systematic review of meta-analyses of clinical trials, *British journal of sports medicine*. 2018; 52(20): 1311. doi: 10.1136/bjsports-2017-098285.

93. Segal R., Zwaal C., Green E., et al. Exercise for people with cancer: a systematic review, *Current oncology*. 2017; 24(4): e290-e315. doi: 10.3747/co.24.3619.

94. Maddocks M., Mockett S., and Wilcock A. Is exercise an acceptable and practical therapy for people with or cured of cancer? A systematic review, *Cancer treatment reviews*. 2009; 35(4): 383-390. doi: 10.1016/j.ctrv.2008.11.008.

95. Lee J. Effects of Exercise Interventions on Breast Cancer Patients During Adjuvant Therapy: A Systematic Review and Meta-analysis of Randomized Controlled Trials, *Cancer nursing*. 2018. doi: 10.1097/NCC.0000000000000682.

96. Herrstedt A., Bay M. L., Simonsen C., et al. Exercise-mediated improvement of depression in patients with gastro-esophageal junction cancer is linked to kynurenine metabolism, *Acta Oncologica*. 2019; 1-9. doi: 10.1080/0284186X.2018.1558371.

97. Hilfiker R., Meichtry A., Eicher M., et al. Exercise and other non-pharmaceutical interventions for cancer-related fatigue in patients during or after cancer treatment: a systematic review incorporating an indirect-comparisons meta-analysis, *British journal of sports medicine*. 2018; 52(10): 651-658. doi: 10.1136/bjsports-2016-096422.

98. Scott J. M., Zabor E. C., Schwitzer E., et al. Efficacy of Exercise Therapy on Cardiorespiratory Fitness in Patients With Cancer: A Systematic Review and Meta-Analysis, *Journal of clinical oncology : official journal of the American Society of Clinical Oncology*. 2018; 36(22): 2297-2305. doi: 10.1200/JCO.2017.77.5809.

99. Bourke L., Smith D., Steed L., et al. Exercise for Men with Prostate Cancer: A Systematic Review and Meta-analysis, *European urology*. 2016; 69(4): 693-703. doi: 10.1016/j.eururo.2015.10.047.

100. Scott J. M., Nilsen T. S., Gupta D., and Jones L. W. Exercise Therapy and Cardiovascular Toxicity in Cancer, *Circulation*. 2018; 137(11): 1176-1191. doi: 10.1161/CIRCULATIONAHA.117.024671.

101. Li X., Li S., Yan S., et al. Impact of preoperative exercise therapy on surgical outcomes in lung cancer patients with or without COPD: a systematic review and meta-analysis, *Cancer management and research*. 2019; 111765-1777. doi: 10.2147/CMAR.S186432.

102. Vermillion S. A., James A., Dorrell R. D., et al. Preoperative exercise therapy for gastrointestinal cancer patients: a systematic review, *Systematic reviews*. 2018; 7(1): 103. doi: 10.1186/s13643-018-0771-0.

103. Li S., Zhou K., Che G., et al. Enhanced recovery programs in lung cancer surgery: systematic review and meta-analysis of randomized controlled trials, *Cancer management and research*. 2017; 9657-670. doi: 10.2147/CMAR.S150500.
